# Zymosan-A promotes the regeneration of intestinal stem cells by upregulating ASCL2

**DOI:** 10.1038/s41419-022-05301-x

**Published:** 2022-10-20

**Authors:** Jicong Du, Lan Fang, Jianpeng Zhao, Yike Yu, Zhenlan Feng, Yuedong Wang, Ying Cheng, Bailong Li, Fu Gao, Cong Liu

**Affiliations:** Department of Radiation Medicine, Faculty of Naval Medicine, Naval Medical University, 800 Xiangyin Road, 200433 Shanghai, P.R. China

**Keywords:** Experimental models of disease, Drug development, Gastroenteritis

## Abstract

Intestinal stem cells (ISCs) are responsible for intestinal tissue homeostasis and are important for the regeneration of the damaged intestinal epithelia. Through the establishment of ionizing radiation (IR) induced intestinal injury model, we found that a TLR2 agonist, Zymosan-A, promoted the regeneration of ISCs in vivo and in vitro. Zymosan-A improved the survival of abdominal irradiated mice (81.82% of mice in the treated group vs. 30% of mice in the PBS group), inhibited the radiation damage of intestinal tissue, increased the survival rate of intestinal crypts and the number of ISCs after lethal IR in vivo. Through organoid experiments, we found that Zymosan-A promoted the proliferation and differentiation of ISCs after IR. Remarkably, the results of RNA sequencing and Western Blot (WB) showed that Zymosan-A reduced IR-induced intestinal injury via TLR2 signaling pathway and Wnt signaling pathway and Zymosan-A had no radioprotection on TLR2 KO mice, suggesting that Zymosan-A may play a radioprotective role by targeting TLR2. Moreover, our results revealed that Zymosan-A increased ASCL2, a transcription factor of ISCs, playing a core role in the process of Zymosan-A against IR-induced intestinal injury and likely contributing to the survival of intestinal organoids post-radiation. In conclusion, we demonstrated that Zymosan-A promotes the regeneration of ISCs by upregulating ASCL2.

## Introduction

The intestinal epithelium ensures the intestinal to achieve important functions such as absorptive, secretory, and barrier functions [1]. The intestinal epithelium is renewed approximately every 5 days and this renewal is accomplished by intestinal stem cells (ISCs) located at the base of the crypts [2]. ISCs are intermingled with Paneth cells and marked by the expression of several markers such as Lgr5. By using Lgr5-EGFP-IRES-creERT2 mice, researchers have found that Lgr5^+^ ISCs are responsible for intestinal tissue homeostasis and are important for the regeneration of the damaged intestinal epithelia [3]. ISCs are activated rapidly in response to stressful insults, allowing rapid and efficient restoration of the damaged epithelium [4]. This response can be observed upon infection with a pathogen, as well as damage induced by IR [5, 6]. Intestinal tissue is extremely sensitive to IR and exposure to high doses of IR can cause severe intestinal injury and the death of ISCs. We established mice and intestinal organoids models to explore the mechanism of ISCs regeneration after IR.

Toll-like receptors (TLRs), as one of the major families of patterns, recognize receptors (PRRs) and play significant roles in the immune system process and inflammatory response [7, 8]. Recent Studies showed that the functions of TLR2/4/5/8 are involved in the pathogenesis of various intestinal diseases such as inflammatory bowel disease and colorectal cancer in vivo [9, 10]. The study of TLRs and intestinal homeostasis has brought a new perspective to solve the problem of IR-induced intestinal damage. Matthew A Ciorba et al. reported that Lactobacillus probiotic protects intestinal epithelium from radiation injury in a TLR2/COX2-dependent manner [11]. Our team also demonstrated that the activation of TLR4 by monophosphoryl lipid A (MPLA) played a role in intestinal radioprotection [12]. These studies remind us that targeting TLRs can prevent and treat IR-induced intestinal injury. Zymosan-A, which is extracted from the cell wall of yeast (Saccharomyces cerevisiae), has been demonstrated as a potent ligand of TLR2 [13].

In this study, we found that Zymosan-A can upregulate the expression of ASCL2 and promote the regeneration of ISCs and activate TLR2 signaling pathway and WNT signaling pathway, resulting in mitigated IR-induced intestinal injury and improved mouse survival. In addition, Zymosan-A might be a potentially highly effective and selective intestinal radioprotective agent.

## Materials and methods

### Chemicals and reagents

Zymosan-A was purchased from Sigma Aldrich Corp (St. Louis, MO, USA), WR2721 and normal saline (NS) was obtained from ChangHai Hospital (Shanghai, China). The PCR kit (RR036A and RR420A) was purchased from TAKARA (Japan). PBS, RPMI 1640, DMEM, and fetal bovine serum (FBS) were supplied by Gibco (New York, USA). IntestiCult™ Organoid Growth Medium was obtained from STEM CELL (Canada). The antibodies for Western blot (GAPDH, YAP1, WNT5A, WNT3A, MYD88, TLR2, OLFM4, ASCL2, CYCLIND1, AXIN2) were purchased from Cell Signaling Technology (Massachusetts, USA). The antibody for western blot (ASCL2) was purchased from Biorbyt (Cambridge, United Kingdom). In Situ Cell Death Detection Kit was obtained from Roche (Basel, Switzerland). Small molecule Foscenvivint (ICG-001) was purchased from Selleck. The primes were obtained from Shenggong Biotech (Shanghai, China).

**Table:** 

LGR5- Forward Primer	CCTACTCGAAGACTTACCCAGT
LGR5- Reverse Primer	GCATTGGGGTGAATGATAGCA
ASCL2- Forward Primer	AAGCACACCTTGACTGGTACG
ASCL2- Reverse Primer	AAGTGGACGTTTGCACCTTCA

### Animals and treatment

Male and female C57BL/6 mice, aged 6–8 weeks old, were obtained from China Academy of Science (Shanghai, China). TLR2 KO mice and Lgr5-EGFP-IRES-creERT2 mice (JAX stock #008875, RRID: IMSR_JAX:008875), aged 6–8 weeks old, were purchased from Cyagen (Jiangsu, China). All mice were housed in a laboratory animal room under standard conditions. The experiments were approved by the Laboratory Animal Center of the Naval Medical University, China in conformance with the National Institute of Health Guide for the Care and Use of Laboratory Animals. We used WR2721 as the positive agent and PBS as the negative agent. The mice were treated with Zymosan-A (25.0 mg/Kg, dissolved in NS) or PBS (200 μl/mice) via peritoneal injection 24 and 2 h before IR, WR2721 (360.0 mg/kg) was used as a positive agent.

### Irradiation

^60^Co (Naval Medical University, China) was used to irradiate the mice and the intestinal organoids at room temperature. The mice were irradiated at 8.0, 8.5, 9.5, or 12.0 Gy to establish the Total Body Irradiation (TBI) model. In order to establish the Abdominal Irradiation (ABI) model, mice hind limbs were shielded with the lead plate to avoid hematopoietic cell death before 20.0 Gy IR exposure.

### Histological examination

Small intestinal tissues were collected from mice and then fixed in 4% paraformaldehyde after IR. Hematoxylin and Eosin (HE), TUNEL (Terminal deoxynucleotidyl transferase dUTP nick-end labeling) staining, Ki-67 staining, and BrdU staining, and Phloxine staining were performed according to the manufacturer’s instructions. The TUNEL^+^ cells were counted in 10 crypts per section. The Ki-67-positive area per section was measured using ImageJ software (National Institutes of Health, Bethesda, MD, USA).

### Fluorescence in situ hybridization (FISH)

Lgr5 FISH was used to detect the level of Lgr5 transcripts in mice intestines. The small intestine was removed from mice and then fixed in 4% paraformaldehyde after IR. The Lgr5 FISH probe (Sequence: 5‘-CY3-GACGACAGGCGGTTGGACGATAGGT-CY3-3’) is designed to hybridize to the LGR5 gene. Lgr5^+^ FISH was conducted according to the manufacturer’s instructions. Fluorescence microscopy was used to observe FISH results.

### Intestine Immunofluorescence

Immunofluorescence analysis was used to detect the expression of OLFM4. The intestinal tissues or intestinal organoid was fixed in 4% paraformaldehyde for 25 min and permeabilized in 0.5% Triton X-100 for 10 min. After blocking in BSA, the intestinal tissues or intestinal organoid was stained with antibodies, followed by the secondary antibody (1:1000). The images were obtained with a fluorescent microscope.

### Intestinal organoid culture

The small intestine was removed from the mice and rinsed with cold PBS after a longitudinal incision. The villi were gently scraped and the remaining tissue was washed ~10 times with cold PBS. The tissue was cut into 2–3 mm fragments, transferred to 15 mM EDTA/PBS, and incubated at 4 °C for 1 h. After incubation, the tissue fragments were shaken vigorously and pelleted approximately three times with cold PBS at 290 rpm for 5 min. Then isolated crypts were embedded in Matrigel (Corning, New York, USA) and cultured in a crypt culture medium (IntestiCult™ Organoid Growth Medium, STEM CELL, Canada). The intestinal organoid culture medium was changed every 3 days.

For radiation experiments, mouse intestinal isolated crypts suspended in Matrigel (250 crypts/50 μl per well) were placed at the center of each well of 24-well plates. Next, 500 μl organoid growth medium was dispensed to each well. Organoids were irradiated 24 h after inoculation. Next, Zymosan-A was added to the cells 12 and 2 h before IR. The mature organoids were observed under a microscope on day 5 after IR. For radiation response assays, the surface area and budding situation of the organoids were measured using Image J software (National Institutes of Health, Bethesda, MD, USA). Lgr5^+^ FISH, OLFM4 Immunofluorescence, and Ki-67 staining were used to observe the organoids.

### RNA sequencing and functional enrichment analysis

Total RNA was isolated from the intestine of mice using Trizol (Invitrogen, USA) at 24 h after radiation. NanoVue (GE, USA) was used to assess RNA purity. Each RNA sample had an A260:A280 ratio greater than 1.8 and an A260:A230 ratio greater than 2.0. Sequencing was performed at Oebiotech (Shanghai, China) with the Illumina HiSeq 2500 system. Prior to sequencing, the raw data were filtered to produce high-quality clean data. All the subsequent analyses were performed using clean data. All the differentially expressed genes were used for heatmap analysis, Gene Ontology Analysis, and KEGG ontology enrichment analyses.

### Western Blot

Total protein from the intestine was extracted using a mammalian protein extraction reagent according to the manufacturer’s protocol and then analyzed by western blotting to detect GAPDH, TLR2, MYD88, YAP1, WNT5A, WNT3A, OLFM4, ASCL2, CYCLIND1, and AXIN2. The secondary antibody (1:1000) was also purchased from Cell Signaling Technology.

### Statistical analysis

Data were expressed as means ± the standard errors of means. Two-tailed Student’s *t*-test was used to analyze the differences between two groups. One-way ANOVA was employed to analyze the differences among three groups. Kaplan–Meier analysis was applied to estimate the difference in overall survival between two groups. The data were analyzed using SPSS ver. 19 software (IBM Corp, Armonk, New York, USA). *P* < 0.05 was considered statistically significant.

## Results

### Zymosan-A mitigated IR-induced intestinal injury in mice

**Fig. 1:**
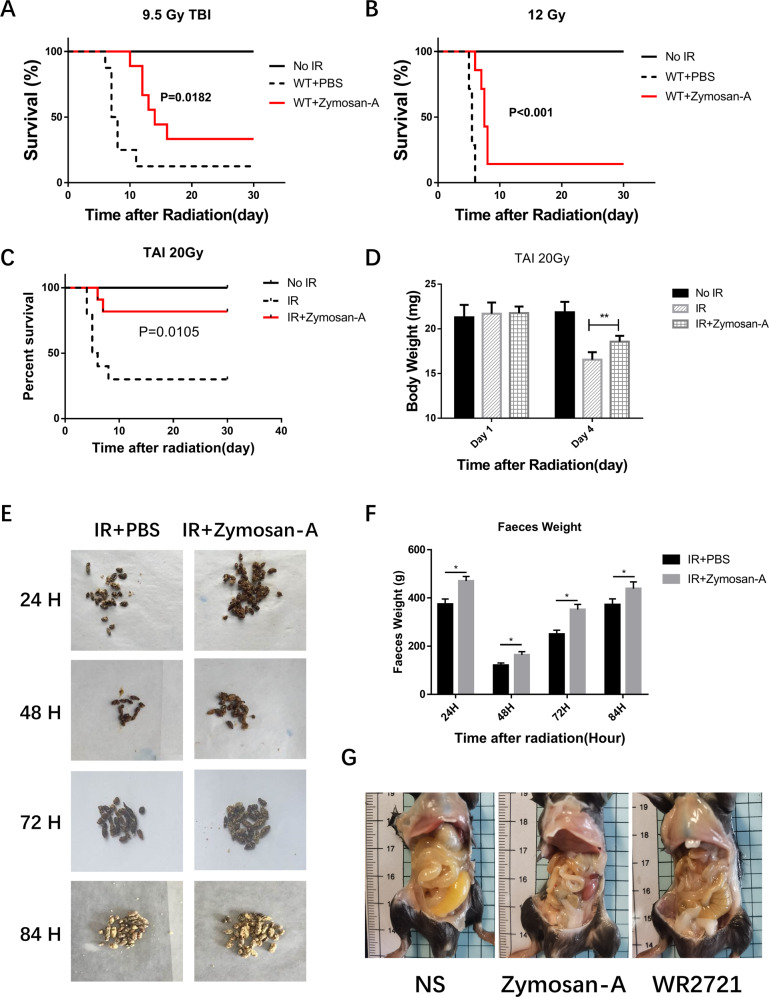
Zymosan-A showed a significant radioprotective effect in vivo. **A**–**C** Male mice were treated with Zymosan-A 25.0 mg/kg, dissolved in NS) by peritoneal injection at 24 h and 2 h before IR, and then exposed to 9.5 Gy, 12.0 Gy TBI, and 20.0 Gy ABI. Control mice were treated with PBS. Survival was recorded. **D** The body weight of mice after 20.0 Gy ABI was recorded. **E** The changes of mouse stool quality at different time points after IR. **F** Statistics of mouse stool quality. **G** Gross pathological view of mouse intestine 84 h after IR. The data were presented as mean ± SD. *P < 0.05 and **P < 0.01 for control versus Zymosan-A treatment.

In our previous work, we showed that the radioprotective effect of Zymosan-A is even better when Zymosan-A was given 24 and 2 h before IR. To find out the best dose of Zymosan-A, we did the dose-response analysis of Zymosan-A, and WR2721 (360 mg/kg) was used as the positive agent. The dose-response curve of Zymosan-A revealed that the optimal dose of the Zymosan-A is 25.0 mg/kg at 24 and 2 h before IR (Fig. S1A). To explore the roles of Zymosan-A in the process of ISCs regeneration in vivo, C57BL/6 mice were treated with Zymosan-A (25.0 mg/kg) intraperitoneally at 24 h and 2 h before IR. The negative control mice were treated with the same amount of PBS. Then mice were exposed to 9.5 Gy and 12.0 Gy total body irradiation (TBI). The results showed that the survival rates of Zymosan-A group were increased after 9.5 and 12.0 Gy TBI (Fig. 1A, B). The TBI results proved the radioprotection of Zymosan-A. Then we established the 20.0 Gy abdominal irradiation (ABI) model, and we found that Zymosan-A protected 81.82% of the mice from IR-induced death, while only 30% of control mice survived beyond 30 days (Fig. 1C). These survival results showed that Zymosan-A could significantly improve the 30-day survival rate and prolong the average survival time of irradiated mice under TBI and ABI. Regretfully, we found that Zymosan-A had a weak therapeutic effect on mice after irradiation by the established mice TBI model (Fig. S1B). In addition, Zymosan-A also had a significant protective effect on the body weight change of mice after 20.0 Gy ABI (Fig. 1D). In order to observe the effect of Zymosan-A on the intestinal function after IR exposure, we recorded the feces of the mice at different time points after 20.0 Gy ABI and found that the fecal quality of the mice in the Zymosan-A treated group was significantly higher than that in PBS group (Fig. 1E, F). Moreover, the gross changes in mice intestines were observed 84 h after IR, which showed that the Zymosan-A could significantly reduce the degree of bleeding and edema in the intestinal tissue (Fig. 1G). Taken together, these results revealed that Zymosan-A has an obvious radioprotective effect on irradiated mice and Zymosan-A mitigated IR-induced intestinal injury in mice.

### Zymosan-A protected the intestinal issue against IR-induced injury

**Fig. 2:**
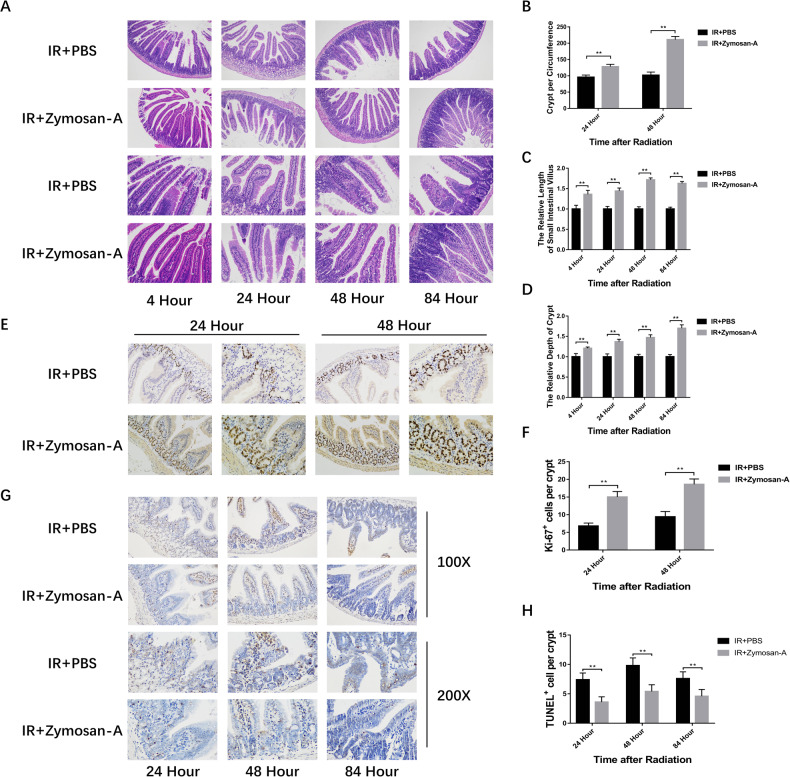
Zymosan-A protected the intestinal issue against IR-induced injury. C57BL/6 mice were intraperitoneally injected with 25 mg/kg/time of Zymosan-A 24 h before and 2 h before 9.5 Gy TBI. **A** Representative images of HE-stained intestinal sections with the indicated treatment at 4, 24, 48, and 84 h. **B** Number of viable crypts. **C** Villus length. **D** Depth of viable crypts. **E** Representative images of Ki-67 immunohistochemical staining intestinal sections with the indicated treatment at 24 and 48 h. **F** The Ki-67^+^ cells per crypts. **G** Representative images of TUNEL immunohistochemical staining intestinal sections with the indicated treatment at 24, 48, and 84 h. **H** The TUNEL^+^ cells per crypts. The data were presented as mean ± SD. *P < 0.05, **P < 0.01, and ***P < 0.001 for control versus Zymosan-A treatment.

Subsequently, the small intestine of mice was collected after IR to evaluate the degree of intestinal injury. C57BL/6 mice were treated with Zymosan-A (25.0 mg/kg) intraperitoneally at 24 and 2 h before IR. The control mice were treated with the same amount of PBS. Then mice were exposed to 9.5 Gy TBI. At 4, 24, 48, and 84 h after 9.5 Gy TBI. The small intestine of mice was taken for HE staining. HE staining results showed that Zymosan-A could improve the intestinal integrity of mice after IR (Fig. 2A). The number of crypts per circumference (Fig. 2B), the villi length (Fig. 2C), and the depth of crypts (Fig. 2D) in Zymosan-A treated group was better than that in the control group. Microcolony formation assay serves as a surrogate for ISCs survival. Ki-67 immunohistochemistry was applied to help visualize the microcolony formation assay and the results of Ki-67 showed that Zymosan-A group had more Ki-67^+^ cells in crypts at 24 and 48 h after IR, which means that Zymosan-A could promote the regeneration of ISCs (Fig. 2E, F). Moreover, BUDR staining was also preformed and BUDR staining results showed that Zymosan-A could promote the regeneration of ISCs (Fig. S1D). In addition, TUNEL staining was used to assay the apoptosis of crypts showing that Zymosan-A could significantly inhibit the apoptosis of intestinal crypts after IR (Fig. 2G, H). These results proved that Zymosan-A had great radioprotective effects on IR-induced intestinal injury.

### Zymosan-A promoted the regeneration of ISCs after IR

**Fig. 3:**
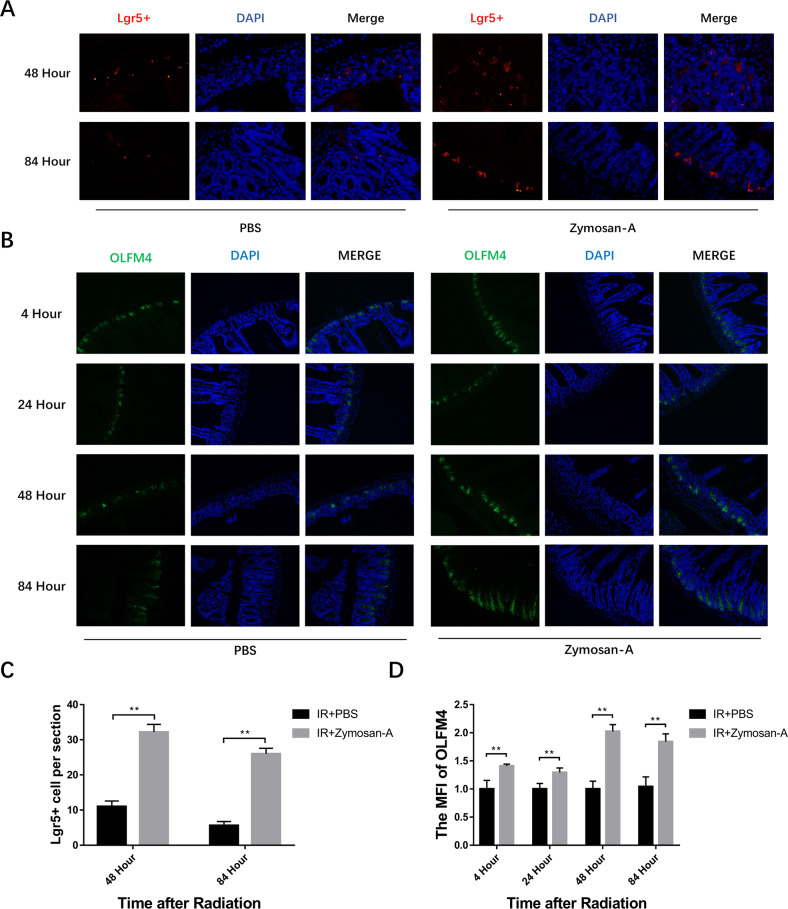
Zymosan-A promoted the regeneration of ISCs after IR. **A** The representative images of Lgr5^+^ FISH intestinal sections with the indicated treatment at 48 and 84 h after TBI. **B** The representative images of OLFM4 immunofluorescences intestinal sections with the indicated treatment at 4, 24, 48, and 84 h after TBI. **C** The Lgr5^+^ cells per section. **D** The OLFM4^+^ cells per section. The data were presented as mean ± SD. *P < 0.05 and **P < 0.01 for control versus Zymosan-A treatment.

ISCs are responsible for intestinal tissue homeostasis and are important for the regeneration of the damaged intestinal epithelia. The Ki-67 result suggested Zymosan-A could promote the regeneration of ISCs. Next, we focused on the regeneration of ISCs after being treated with Zymosan-A or PBS. Lgr5 (leucine-rich-repeat-containing G-protein-coupled receptor 5) is both a marker of adult ISCs and a critical modulator of their activity via its role as an effector of Wnt signaling [14]. As a marker of ISCs, the Lgr5^+^ crypt base columnar cells generate all epithelial lineages, suggesting that it represents the genuine stem cell of the small intestine [1]. Lgr5^+^ ISCs has great major advances in the process of stem cell biology during homeostasis, regeneration, and disease [15]. To determine whether Zymosan-A acted directly on ISCs, Lgr5^+^ fluorescence in situ hybridization experiments (FISH) were conducted. The surviving ISCs in the intestinal crypts at 48 and 84 h after IR with or without Zymosan-A treatment were evaluated and the Lgr5^+^ FISH result showed that IR could significantly reduce the number of Lgr5^+^ ISCs, while Zymosan-A significantly increased the number of Lgr5^+^ ISCs (Fig. 3A, C). These data proved that Zymosan-A exerted a protective effect on IR-induced ISCs injury.

Furthermore, we applied another ISCs marker, OLFM4, to investigate the mitigation effect of Zymosan-A on IR-induced ISCs injury. OLFM4 is expressed by intestinal epithelial cells residing at the base of the crypt, including the crypt base columnar cells. The numbers and functions of ISCs were assessed by analyzing OLFM4^+^ ISCs. The results of OLFM4 immunofluorescences showed that Zymosan-A could significantly increase the number of OLFM4^+^ ISCs in the intestine of irradiated mice (Fig. 3B, D). In addition, Phloxine staining was preformed to evaluate the number of Paneth cells and we found that there were no changes in the number of Paneth cells between Zymosan-A treated group and the control group (Fig. S2). Taken together, Zymosan-A could significantly increase the number of Lgr5^+^ ISCs and OLFM4^+^ ISCs in the intestinal tract of irradiated mice, suggesting that Zymosan-A may play a radioprotective effect by promoting the regeneration of ISCs after IR.

### Zymosan-A protected the intestinal organoid against IR-induced injury

**Fig. 4:**
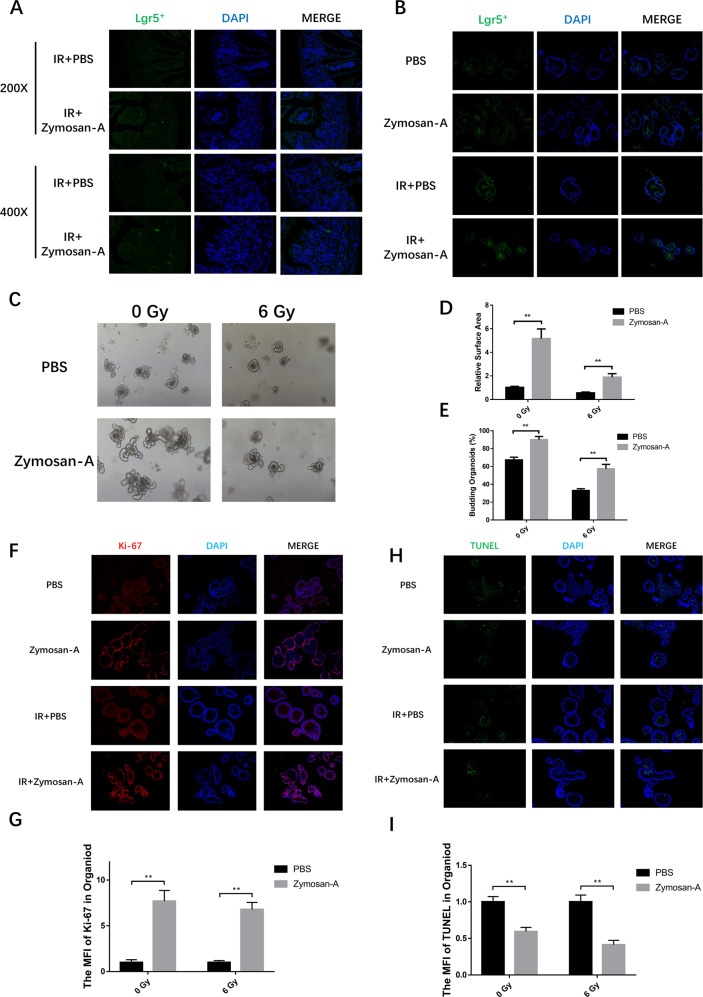
Zymosan-A protected the intestinal organoid against IR-induced injury. **A** Intestine of Lgr5-EGFP-IRES-creERT2 mice (JAX:008875) were extracted and Lgr5 fluorescence were conducted. **B** Intestinal crypts of (JAX:008875) were extracted for organoid culture, and then it was stimulated with Zymosan-A (25.0 μg/ml) or PBS 12 and 2 h before 6.0 Gy IR. Then Lgr5 fluorescence were conducted. **C** Intestinal crypts of C57BL/6 mice were extracted for organoid culture, and then it was stimulated with Zymosan-A (25.0 μg/ml) or PBS 12 and 2 h before 6.0 Gy IR. Organoid regeneration after IR with PBS and Zymosan-A treatment. **D** The relative area of intestinal organoids. **E** The percent of budding intestinal organoids. **F** The expression of the Ki-67 was detected by fluorescence microscopy. **G** The MFI of Ki-67 in intestinal organoids. **H** The expression of the TUNEL was detected by fluorescence microscopy. **I** The MFI of TUNEL in intestinal organoids. The data were presented as mean ± SD. *P < 0.05, **P < 0.01, and ***P < 0.001 for control versus Zymosan-A treatment.

To better analyze the regeneration of ISCs after IR, we used Lgr5-EGFP-IRES-creERT2 mice as a way to quantitate Lgr5^+^ ISCs. Lgr5-EGFP-IRES-creERT2 mice were treated with Zymosan-A or PBS before IR. Then the intestine fluorescence analysis was used to detect the expression of Lgr5. This result showed that IR could significantly reduce the number of Lgr5^+^ ISCs, while Zymosan-A significantly increased the number of Lgr5^+^ ISCs (Fig. 4A). Intestinal organoids are three-dimensional spheroids with an intact gut epithelial structure and contain all types of enteric epithelial functional cells, including intestinal epithelial cells, enteroendocrine cells, goblet cells, Paneth cells and Lgr5^+^ ISCs [16]. The intestinal organoid is a great technology to study the proliferation, differentiation, and regeneration of ISCs in vitro. Intestinal organoid culturing has also been applied to study the pathophysiology of intestinal diseases [17].

In this work, Intestinal organoids were also used to explore the regeneration of ISCs after Zymosan-A treatment. Intestinal crypts of C57BL/6 mice were extracted for organoid culture, and then it was stimulated with Zymosan-A (25.0 μg /ml) or PBS 12 and 2 h before 6.0 Gy IR. The changes in organoids were observed after 7 days. Crypts were extracted from Lgr5-EGFP-IRES-creERT2 to culture intestinal organoids. As shown in Fig. 4B. Lgr5 fluorescence was also conducted in intestinal organoids and results showed that Zymosan-A also increased the number of Lgr5+ ISCs (Fig. 4B). Moreover, the results of intestinal organoids from wild mice showed that Zymosan-A could improve the ability of organoid formation (Fig. 4C). Compared with the PBS group, the relative volume of single intestinal organoid in the Zymosan-A group was increased (Fig. 4D). And the percent of budding intestinal organoids was increased in the Zymosan-A group (Fig. 4E). Ki-67 and TUNEL staining were also performed on the intestinal organoids after 7 days. We found that the MFI of Ki-67 was increased in Zymosan-A (Fig. 4F, G), and the MFI of TUNEL was decreased in Zymosan-A group. These data suggested that Zymosan-A could significantly promote the proliferation and inhibit apoptosis of the intestinal organoids after IR (Fig. 4H, I). These results suggested that Zymosan-A could significantly improve the proliferation and differentiation of irradiated ISCs.

### Zymosan-A promoted ISCs regeneration via TLR2 signaling pathway and Wnt signaling pathway

**Fig. 5:**
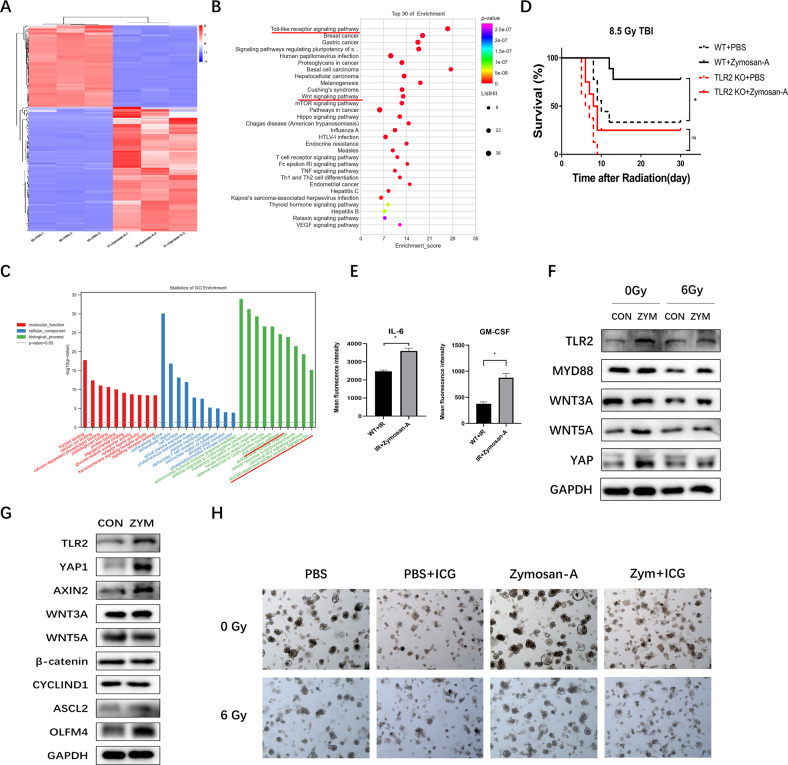
Zymosan-A promoted the regeneration of ISCs via TLR2 signaling pathway and Wnt signaling pathway. **A** Heatmap of DEGs between IR + PBS mice and IR + Zymosan-A mice. **B** Pathway enrichment analysis of KEGG. **C** GO term analysis was performed on DEGs. **D** C57BL/6 mice and TLR2 KO mice were intraperitoneally injected with Zymosan-A (25.0 mg/kg) 24 and 2 h before 8.5 Gy TBI, Control mice were treated with PBS. Survival was recorded. **E** The levels of IL-6 and GM-CSF in the serum of mice pretreated with Zymosan-A or PBS at 24 h after IR. **F** WB analysis of intestinal tissue proteins. **G** WB analysis of intestinal organoid proteins; **H** Representative images of intestinal organoids with ICG-001 (25.0 μM) treatment.The data were presented as mean ± SD. *P < 0.05, **P < 0.01, and ***P < 0.001 for control versus Zymosan-A treatment.

RNA-sequence (RNA-seq) technology was performed to elucidate the mechanism of Zymosan-A mediated ISCs regeneration. The intestinal tissues of mice were taken for RNA-seq at 24 h after 9.5 Gy TBI. We screened out differentially expressed genes (DEGs) and performed secondary analysis, and a total of 169 DEGs were screened out by the secondary analysis (Supplementary Table S1), including 102 upregulated DEGs and 67 downregulated DEGs, and cluster analysis was conducted on the DEGs (Fig. 5A). Then we performed KEGG and GO analyses to elucidate the biological functions of DEGs. KEGG pathway enrichment analysis showed that DEGs were significantly enriched in MMU04620: Toll-like receptor signaling Pathway, MMU04310: Wnt Signaling Pathway, and MMU04150: MTOR Signaling Pathway, MMU04390: Hippo Signaling Pathway, etc. (Fig. 5B) (Supplementary Table S2). GO functional enrichment analysis also found that GO0016055: Wnt signaling Pathway and GO0002755: MyD88-Dependent Toll-like receptor signaling Pathway were significantly enriched (Fig. 5C) (Supplementary Table S3). RNA-Seq results indicated that Zymosan-A may promote the regeneration of ISCs by activating the Toll-like receptor signaling pathway and Wnt signaling pathway.

Zymosan-A has been demonstrated as a potent ligand of TLR2. In our previous study, we found that TLR2 had a critical role in radioresistance in vivo [18]. Compared with WT mice, TLR2 KO mice were more susceptible to radiation-induced death. Furthermore, TLR2 KO mice were used to verify the function of TLR2 in the process of Zymosan-A mediated intestinal radioprotection. The survival date showed that Zymosan-A had no radioprotective effects on TLR2 KO mice (Fig. 5D), suggesting that Zymosan-A played a radioprotective effect by targeting the activation of the TLR2 signaling pathway. In addition, we detected the level of IL-6 and GM-CSF in the serum of mice pretreated with Zymosan-A or PBS, and we found that the serum levels of IL-6 and GM-CSF were upregulated in vivo at 24 h after IR (Fig. 5E).

The KEGG pathway enrichment analysis showed that DEGs were significantly enriched in Wnt Signaling Pathway and Toll-like receptor signaling Pathway. Subsequently, we verified the changes of key proteins in Toll-like receptor signaling Pathway in intestinal tissues. The WB result showed that Zymosan-A significantly upregulated the expression levels of key molecules such as TLR2, MyD88 with or without IR exposure (Fig. 5F), which was consistent with the previous RNA-Seq (Fig. 5B, C). Moreover, the expression levels of key molecules in Wnt signaling Pathway and Toll-like receptor signaling Pathway were also evaluated in intestinal organoids. By using WB, we found that TLR2, Wnt 3a, β-catenin, OLFM4, and ASCL2 were increased (Fig. 5G). We used Foscenvivint (ICG-001), a selective small molecule inhibitor of CBP/β-catenin complex formation, to inhibit the Wnt Signaling Pathway. Compared with the Zymosan-A treated organoids, the Zymosan-A + ICG-001 treated organoids were more susceptible to radiation-induced deaths (Fig. 5H). Zymosan-A protected PBS-treated organoids from radiation-induced death but had no radioprotective effects on the ICG-001 treated organoids (Fig. 5H). These findings consistently indicated that Zymosan-A induced radioprotective effects via Wnt Signaling Pathway. Taken together, the RNA-Seq and TLR2 KO mice results suggested that Zymosan-A may promote the regeneration of ISCs by activating TLR2 signaling pathway and Wnt signaling pathway.

### The intestinal radioprotection of Zymosan-A was ASCL2-dependent

**Fig. 6:**
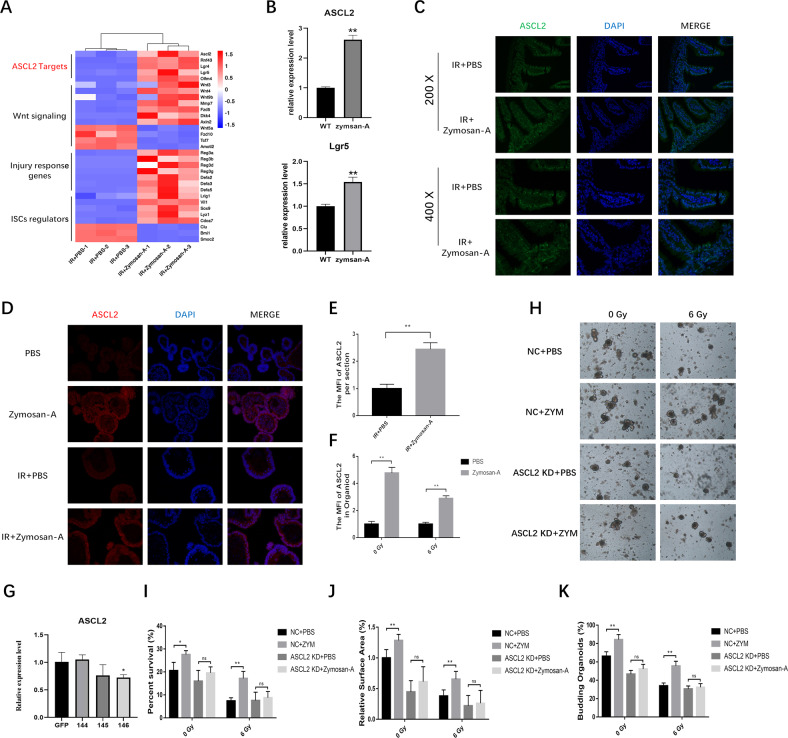
The radioprotection of Zymosan-A in intestinal organoids was ASCL2-dependent. **A** Heatmap of ASCL2 downstream target genes, genes involved in Wnt signaling, and injury response genes, and ISCs regulators in intestine. **B** quantitative PCR results of Lgr5 and ASCL2 in intestinal tissue. **C** The expression of the ASCL2 was detected by fluorescence microscopy in intestine. **D** The expression of the ASCL2 was detected by fluorescence microscopy in intestinal organoids. **E** The MFI of ASCL2 in intestine. **F** The MFI of ASCL2 in intestinal organoids. **G** the relative expression of ASCL2 after shRNA knockdown. **H** The morphology of ASCL2 KD Organoids after IR. **I** The survival rate of intestinal organoids. **J** The ralative surface of intestinal organoids. **K** The budding rate of intestinal organoids. The data were presented as mean ± SD. *P < 0.05, **P < 0.01, and ***P < 0.001 for control versus Zymosan-A treatment.

The RNA-Seq result suggested that Zymosan-A could significantly regulate the expression of several genes related to ISCs and intestinal injury response. Among the genes regulating intestinal stem cells, we found that ASCL2 was significantly upregulated by Zymosan-A (Fig. 6A). ASCL2 (Achaette-scute Homologue 2) is a member of the basic helical ring helical (bHLH) family [19]. As a transcription factor, ASCL2, itself encoded by a crypt-specific Wnt target gene, is a master regulator of intestinal stem cell identity. Ectopic expression of ASCL2 using the intestinal epithelium-specific Villin promoter induces hyper-proliferation of crypts, expansion of the expression domain of the ISCs markers Lgr5 and Sox9, and the formation of hyperproliferative pockets on the villus. This has led to the conclusion that ASCL2 is a master regulator of crypt stemness [20]. As a downstream target of the Wnt pathway, ASCL2 protein upregulates LGR5 and OLFM4. According to the result of RNA-Seq and published research, we try to figure out the function of ASCL2 in the process of Zymosan-A mediated intestinal radioprotection. Then we found that the expression of ASCL2 could be significantly upregulated by Zymosan-A by using QT-PCR (Fig. 6B) and WB (Fig. 5G). The expression of ASCL2 in the irradiated intestine was also assayed by immunofluorescence, and we found that the expression of ASCL2 could be significantly upregulated by Zymosan-A (Fig. 6C, E). Moreover, the expression of ASCL2 in intestinal organoids was evaluated. As shown in Fig. 6D, F, Zymosan-A could significantly upregulate the expression of ASCL2 in intestinal organoids after IR.

To further explore the role of ASCL2 in the process of Zymosan-A against IR-induced intestinal injury, shRNA was used to knock down (KD) the level of ASCL2 in intestinal organoids. Verified by quantitative PCR, Sequence 146 was used to establish ASCL2 KD intestinal organoids (Fig. 6G). As shown in Fig. 6H, ASCL2 KD Organoids have altered morphology and are more sensitive to IR, and the intestinal radioprotection of Zymosan-A was significantly inhibited after ASCL2 KD. The survival rate, size, and budding rate of intestinal organoids significantly decreased in ASCL2 KD group (Fig. 6I–K). The results of organoids showed that ASCL2 plays a central role in the process of Zymosan-A mediated ISCs regeneration, and ASCL2 may be a potential new therapeutic target. Taken together, these results suggest that Zymosan-A promotes the regeneration of ISCs by upregulating ASCL2.

## Discussion

The continuous regeneration of intestinal epithelium emphasizes the vital role of ISCs in the intestinal tissue, which requires a tightly regulated balance between ISCs proliferation and differentiation [21]. ISCs located at the crypt base migrate upward and differentiate into all the different types of mature epithelial cells to maintain the homeostasis of the intestinal epithelium under steady conditions [22, 23]. Since intestinal epithelial cells directly contact pathogenic environmental factors, ISCs are susceptible to epithelial damage induced by chemicals, pathogens, or irradiation [24–26]. The intestine must respond positively when subjected to stressful insults like IR and pathogens to maintain epithelial integrity. ISCs are much radiosensitive. The common outcome of IR-induced intestinal damage is the loss of Lgr5^+^ ISCs [4, 27]. However, there is more than one type of intestinal stem cell that plays an important role in epithelial damage. When Lgr5^+^ ISCs are depleted, Bmi1^+^ cells localized around the +4 position possess ISCs properties and rapidly revert to Lgr5^+^ ISCs to sustain intestinal homeostasis [28]. These re-emerged Lgr5^+^ stem cells have been proven to be essential for post-radiation epithelial repair because Lgr5^+^ ISCs are required for post-radiation epithelial recovery [29].

As sensors of microbial infection, TLRs are critical for the initiation of immune defense responses and inflammatory [30, 31]. In recent years, research has been reported about TLRs playing a critical role in radiation protection [11, 32, 33]. Zymosan-A, an extract of the yeast cell wall, has been proven to protect against radiation-induced hematopoietic damage by targeting TLR2 in our previous study [18]. In this study, we found that Zymosan-A exhibited significant radioprotective effects on IR-induced intestinal injury in vivo and in vitro. Through animal experiments, we found that Zymosan-A could significantly improve the 30-day survival rate and prolong the average survival time of irradiated mice under TBI and ABI. The gross changes in mice intestines also showed that the Zymosan-A could significantly reduce the degree of bleeding and edema in the intestinal tissue. The results of HE staining, Ki-67 immunohistochemistry, and TUNEL staining revealed that Zymosan-A could significantly promote the regeneration of ISCs and inhibit the apoptosis of intestinal crypts after IR.

ISCs are responsible for intestinal tissue homeostasis and are important for the regeneration of the damaged intestinal epithelia. Over the years, many studies have been carried out on the mechanism and prevention of IR-induced intestinal injury, proving that ISCs play a central role in the process of IR-induced intestinal injury [34]. IR can directly damage ISCs, and impair the proliferation and differentiation of ISCs [35]. Understanding the regulatory mechanisms of ISCs is key to preventing and treating IR-induced intestinal injury. So we focused on the regeneration of ISCs after IR and found that Zymosan-A could significantly increase the number of Lgr5^+^ ISCs and OLFM4^+^ ISCs in the intestinal tract of irradiated mice. To better analyze the regeneration of ISCs after IR, Lgr5-EGFP-IRES-creERT2 mice were used to quantitate Lgr5+ ISCs, and the intestine fluorescence analysis showed that IR could significantly reduce the number of Lgr5+ ISCs, while Zymosan-A significantly increased the number of Lgr5+ ISCs. These results suggested that Zymosan-A may play a radioprotective effect by promoting the regeneration of ISCs after IR.

Intestinal organoids have unlimited proliferation ability and can simulate many characteristics of the intestine [16]. It can accurately simulate the physiological state of the intestinal epithelium and provided a new research approach for ISCs [36]. Through organoid experiments, we found that Zymosan-A improved the ability of crypt organoid formation. Compared with the IR group, the Zymosan-A group had more extensive crypt organoid formation, and the number and volume of single organoid buds were increased. Ki-67 staining and TUNEL staining of intestinal organoids showed that Zymosan-A could promote the proliferation of intestinal organoids and inhibit the apoptosis of intestinal organoids after IR. These results confirmed that Zymosan-A significantly prompted the regeneration of ISCs in intestinal organoids after IR.

To determine the possible mechanism underlying radioprotection by Zymosan-A, we performed RNA-Seq technology to explore the mechanism of Zymosan-A mediated intestinal radioprotection. We screened out 169 DEGs, including 102 upregulated DEGs and 67 downregulated DEGs. The results of KEGG and GO analyses indicated that Zymosan-A may play a radioprotective role in IR-intestinal injury by activating Toll-like receptor signaling pathway and Wnt signaling pathway. Moreover, the survival data showed that Zymosan-A had no radioprotective effects on TLR2 KO mice, and the expression levels of key molecules in Wnt signaling Pathway and TLR2 signaling Pathway were also evaluated in intestinal organoids by using WB. By using ICG-001, a selective small molecule inhibitor of CBP/β-catenin complex formation, we found that Zymosan-A protected PBS-treated organoids from radiation-induced death, but had no radioprotective effects on the ICG-001 treated organoids, which indicated that Zymosan-A induced radioprotective effects via Wnt Signaling Pathway. Taken together, the RNA-Seq and TLR2 KO mice results suggested that Zymosan-A may play a radioprotective role in IR-induced intestinal injury by activating TLR2 signaling pathway and Wnt signaling pathway.

The RNA-Seq result suggested that Zymosan-A could significantly regulate the expression of several genes related to ISCs and intestinal injury response. Among the genes regulating ISCs, we found that ASCL2 was significantly upregulated by Zymosan-A. As a transcription factor, ASCL2 also has been identified as a WNT/β-catenin target gene, and which was shown to be expressed in the mouse intestinal crypt as well as human and mouse intestinal cancers [37]. ASCL2 is a highly restricted fashion in ISCs. Ectopic of ASCL2 in the mouse intestine led to crypt hyperplasia, an expansion in the size of the crypt domain and the formation of ectopic crypts, expansion of the expression domain of the ISCs markers Lgr5 and Sox9, and the formation of hyperproliferative pockets on the villus, whereas genetic deletion of ASCL2 led to a loss of the Lgr5^+^ ISCs. According to the result of RNA-Seq and published research, we try to further explore the role of ASCL2 in the process of Zymosan-A against IR-induced intestinal injury, firstly we used immunofluorescence and found that the expression of ASCL2 in the irradiated intestine and intestinal organoids were evaluated. Moreover, the intestinal radioprotection of Zymosan-A was significantly inhibited after ASCL2 KD. These results suggest that the intestinal radioprotection of Zymosan-A was ASCL2-dependent.

## Conclusion

In conclusion, Zymosan-A can upregulate the expression ASCL2 and promote the regeneration of ISCs by activating TLR2 signaling pathway and WNT signaling pathway, resulting in mitigated IR-induced intestinal injury and improved mouse survival (Fig. S3). Our work demonstrated that Zymosan-A promotes the regeneration of ISCs by up-regulating ASCL2. Zymosan-A may be an effective radioprotective drug for the prevention and treatment of IR-induced intestinal injury.

## Supplementary information

supplementary figure and table legends

Figure S1.

Figure S2.

Figure S3.

Figure S4.

Supplementary Table 1.

Supplementary Table 2.

Supplementary Table 3.

Reproducibility checklist

## Data Availability

All data generated or analyzed during this study are included in this published article and its supplementary information files.

## References

[1] Beumer J, Clevers H (2021). Cell fate specification and differentiation in the adult mammalian intestine. Nat Rev Mol Cell Biol.

[2] Rath E, Moschetta A, Haller D (2018). Mitochondrial function—gatekeeper of intestinal epithelial cell homeostasis. Nat Rev Gastroenterol Hepatol.

[3] Jasper H (2020). Intestinal stem cell aging: origins and interventions. Annu Rev Physiol.

[4] Santos AJM, Lo YH, Mah AT, Kuo CJ (2018). The intestinal stem cell niche: homeostasis and adaptations. Trends Cell Biol.

[5] Xie LW, Cai S, Zhao TS, Li M, Tian Y (2020). Green tea derivative (−)-epigallocatechin-3-gallate (EGCG) confers protection against ionizing radiation-induced intestinal epithelial cell death both in vitro and in vivo. Free Radic Biol Med.

[6] Zou WY, Blutt SE, Zeng XL, Chen MS, Lo YH, Castillo-Azofeifa D (2018). Epithelial WNT ligands are essential drivers of intestinal stem cell activation. Cell Rep.

[7] Mukherjee S, Huda S, Sinha Babu SP (2019). Toll-like receptor polymorphism in host immune response to infectious diseases: a review. Scand J Immunol.

[8] Mangoni M, Sottili M, Gerini C, Desideri I, Bastida C, Pallotta S (2017). A PPAR-gamma agonist protects from radiation-induced intestinal toxicity. U Eur Gastroenterol J.

[9] Lu Y, Li X, Liu S, Zhang Y, Zhang D (2018). Toll-like receptors and inflammatory bowel disease. Front Immunol.

[10] Reilly F, Burke JP, Lennon G, Kay EW, McNamara DA, Cullen G (2021). A case-control study examining the association of smad7 and TLR single nucleotide polymorphisms on the risk of colorectal cancer in ulcerative colitis. Colorectal Dis.

[11] Riehl TE, Alvarado D, Ee X, Zuckerman A, Foster L, Kapoor V (2019). Lactobacillus rhamnosus GG protects the intestinal epithelium from radiation injury through release of lipoteichoic acid, macrophage activation and the migration of mesenchymal stem cells. Gut..

[12] Liu Z, Cao K, Liao Z, Chen Y, Lei X, Wei Q (2020). Monophosphoryl lipid A alleviated radiation-induced testicular injury through TLR4-dependent exosomes. J Cell Mol Med.

[13] de Graaff P, Berrevoets C, Rӧsch C, Schols HA, Verhoef K, Wichers HJ (2021). Curdlan, zymosan and a yeast-derived β-glucan reshape tumor-associated macrophages into producers of inflammatory chemo-attractants. Cancer Immunol Immunother.

[14] Fernandez Vallone V, Leprovots M, Ribatallada-Soriano D, Gerbier R, Lefort A, Libert F (2020). LGR5 controls extracellular matrix production by stem cells in the developing intestine. EMBO Rep.

[15] Kurokawa K, Hayakawa Y, Koike K. Plasticity of intestinal epithelium: stem cell niches and regulatory signals. Int J Mol Sci. 2020;22:357. 10.3390/ijms22010357 PMC7795504 33396437

[16] Serra D, Mayr U, Boni A, Lukonin I, Rempfler M, Challet Meylan L (2019). Self-organization and symmetry breaking in intestinal organoid development. Nature..

[17] Sprangers J, Zaalberg IC, Maurice MM (2021). Organoid-based modeling of intestinal development, regeneration, and repair. Cell Death Differ.

[18] Du J, Cheng Y, Dong S, Zhang P, Guo J, Han J (2017). Zymosan-A protects the hematopoietic system from radiation-induced damage by targeting TLR2 signaling pathway. Cell Physiol Biochem.

[19] Bogutz AB, Oh-McGinnis R, Jacob KJ, Ho-Lau R, Gu T, Gertsenstein M (2018). Transcription factor ASCL2 is required for development of the glycogen trophoblast cell lineage. PLoS Genet.

[20] Basu S, Gavert N, Brabletz T, Ben-Ze’ev A (2018). The intestinal stem cell regulating gene ASCL2 is required for L1-mediated colon cancer progression. Cancer Lett.

[21] Bankaitis ED, Ha A, Kuo CJ, Magness ST (2018). Reserve stem cells in intestinal homeostasis and injury. Gastroenterology..

[22] van Es JH, Wiebrands K, López-Iglesias C, van de Wetering M, Zeinstra L, van den Born M (2019). Enteroendocrine and tuft cells support Lgr5 stem cells on Paneth cell depletion. Proc Natl Acad Sci USA.

[23] Gehart H, Clevers H (2019). Tales from the crypt: new insights into intestinal stem cells. Nat Rev Gastroenterol Hepatol.

[24] Qi Z, Li Y, Zhao B, Xu C, Liu Y, Li H (2017). BMP restricts stemness of intestinal Lgr5(+) stem cells by directly suppressing their signature genes. Nat Commun.

[25] Sheng X, Lin Z, Lv C, Shao C, Bi X, Deng M (2020). Cycling stem cells are radioresistant and regenerate the intestine. Cell Rep.

[26] Giroux V, Stephan J, Chatterji P, Rhoades B, Wileyto EP, Klein-Szanto AJ (2018). Mouse intestinal Krt15+ Crypt cells are radio-resistant and tumor initiating. Stem C ell Rep.

[27] Li M, Gu MM, Lang Y, Shi J, Chen BPC, Guan H (2019). The vanillin derivative VND3207 protects intestine against radiation injury by modulating p53/NOXA signaling pathway and restoring the balance of gut microbiota. Free Radic Biol Med.

[28] Jadhav U, Saxena M, O’Neill NK, Saadatpour A, Yuan GC, Herbert Z (2017). Dynamic reorganization of chromatin accessibility signatures during dedifferentiation of secretory precursors into Lgr5+ intestinal stem cells. Cell Stem Cell.

[29] Ayyaz A, Kumar S, Sangiorgi B, Ghoshal B, Gosio J, Ouladan S (2019). Single-cell transcriptomes of the regenerating intestine reveal a revival stem cell. Nature..

[30] Fitzgerald KA, Kagan JC (2020). Toll-like receptors and the control of immunity. Cell..

[31] Mancini F, Rossi O, Necchi F, Micoli F. OMV Vaccines and the role of TLR agonists in immune response. Int J Mol Sci. 2020;21:4416. 10.3390/ijms21124416 PMC7352230 32575921

[32] Sanguri S, Gupta D (2018). Mannan oligosaccharide requires functional ETC and TLR for biological radiation protection to normal cells. BMC Cell Biol.

[33] Kumar S, Kumar R (2019). Role of acemannan O-acetyl group in murine radioprotection. Carbohydr Polym.

[34] Fu G, Chen S, Liang L, Li X, Tang P, Rao X (2021). SIRT1 inhibitors mitigate radiation-induced GI syndrome by enhancing intestinal-stem-cell survival. Cancer Lett.

[35] Meena SK, Joriya PR, Yadav SM, Kumar R, Meena P, Patel DD. Modulation of radiation-induced intestinal injury by radioprotective agents: a cellular and molecular perspectives. Front Pharmacol. 2022;13:663855. 10.1515/reveh-2021-0108 35438851

[36] Rubert J, Schweiger PJ, Mattivi F, Tuohy K, Jensen KB, Lunardi A (2020). Intestinal organoids: a tool for modelling diet-microbiome-host interactions. Trends Endocrinol Metab.

[37] Murata K, Jadhav U, Madha S, van Es J, Dean J, Cavazza A (2020). Ascl2-dependent cell dedifferentiation drives regeneration of ablated intestinal stem cells. Cell Stem Cell.

