# Supplementary material for: Zymosan-A promotes the regeneration of intestinal stem cells by upregulating ASCL2
Source: Cell Death Dis. 2022 Oct 20;13(10):884. doi: 10.1038/s41419-022-05301-x (PMC9585075; doi:10.1038/s41419-022-05301-x)
Supplement: Supplementary file 1 — supplementary figure and table legends [file 41419_2022_5301_MOESM1_ESM.docx]

**Figure S1. Zymosan-A showed a significant radioprotective effect in female mice.** A. The dose response curve of Zymosan-A. WR2721 (360 mg/kg) was used as the positive agent and PBS as the negative agent. B. The therapeutic effect of Zymosan-A on female mice after IR. The female mice were treated with Zymosan-A (25.0 mg/Kg, dissolved in NS) via peritoneal injection 1 or 24 hours after IR. WR2721 (360 mg/kg) was used as the positive agent and PBS as the negative agent. C. Representative images of HE stained intestinal tissue with the indicated treatment after IR. D. Representative images of BRDU immunofluorescence intestinal tissue with the indicated treatment after IR.

**Figure S2. Zymosan-A had no effect to Paneth cells.** Phloxine staining was preformed to evaluate the number of Paneth cells.

**Figure S3. Schematic diagram of Zymosan-A promotes the regeneration of ISCs.** Zymosan-A can upregulate the expression ASCL2 and promote the regeneration of ISCs and by activating TLR2 signaling pathway and WNT signaling pathway, resulting in mitigated IR-induced intestinal injury and improved mouse survival.

**Figure S4. Full and uncropped western blots.**

**Supplementary Table 1. The list of 169 differentially expressed genes.**

**Supplementary Table 2. The list of KEGG pathway enrichment terms.**

**Supplementary Table 3. The list of GO pathway enrichment terms.**
