# Supplementary figures and images for: Zymosan-A promotes the regeneration of intestinal stem cells by upregulating ASCL2
Source: Cell Death Dis. 2022 Oct 20;13(10):884. doi: 10.1038/s41419-022-05301-x (PMC9585075; doi:10.1038/s41419-022-05301-x)

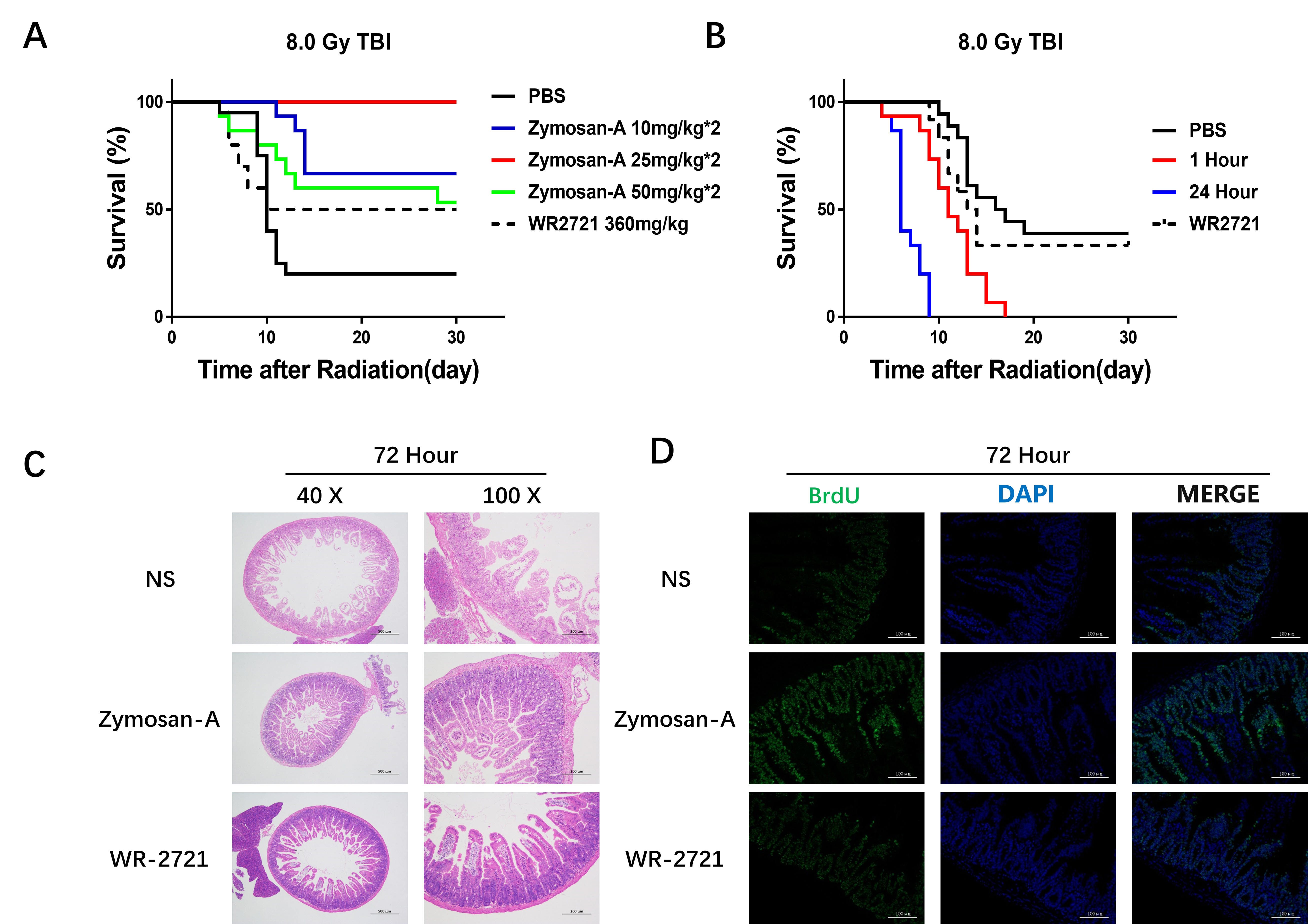

Supplement: Supplementary file 2 — Figure S1. [file 41419_2022_5301_MOESM2_ESM.tif]

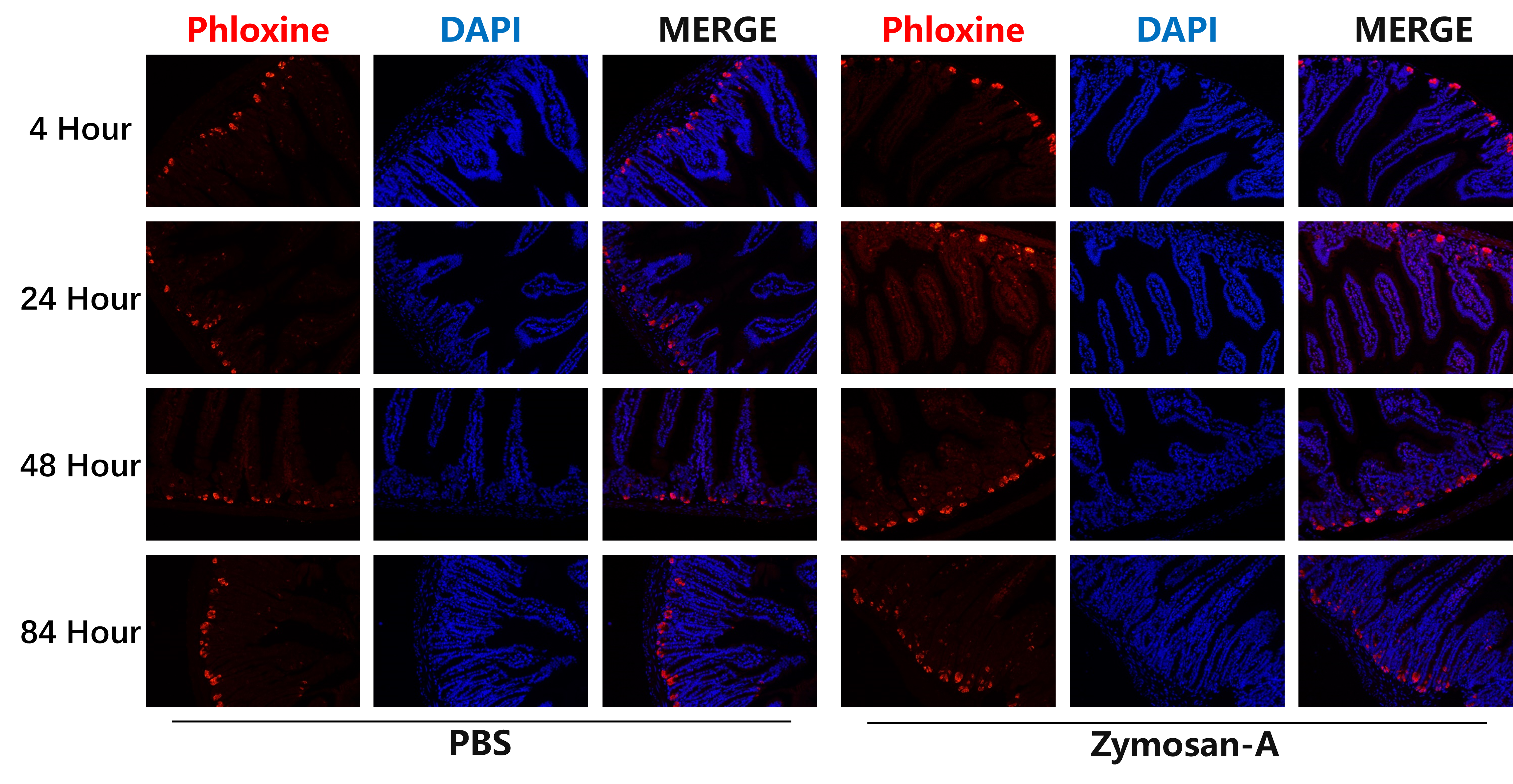

Supplement: Supplementary file 3 — Figure S2. [file 41419_2022_5301_MOESM3_ESM.tif]

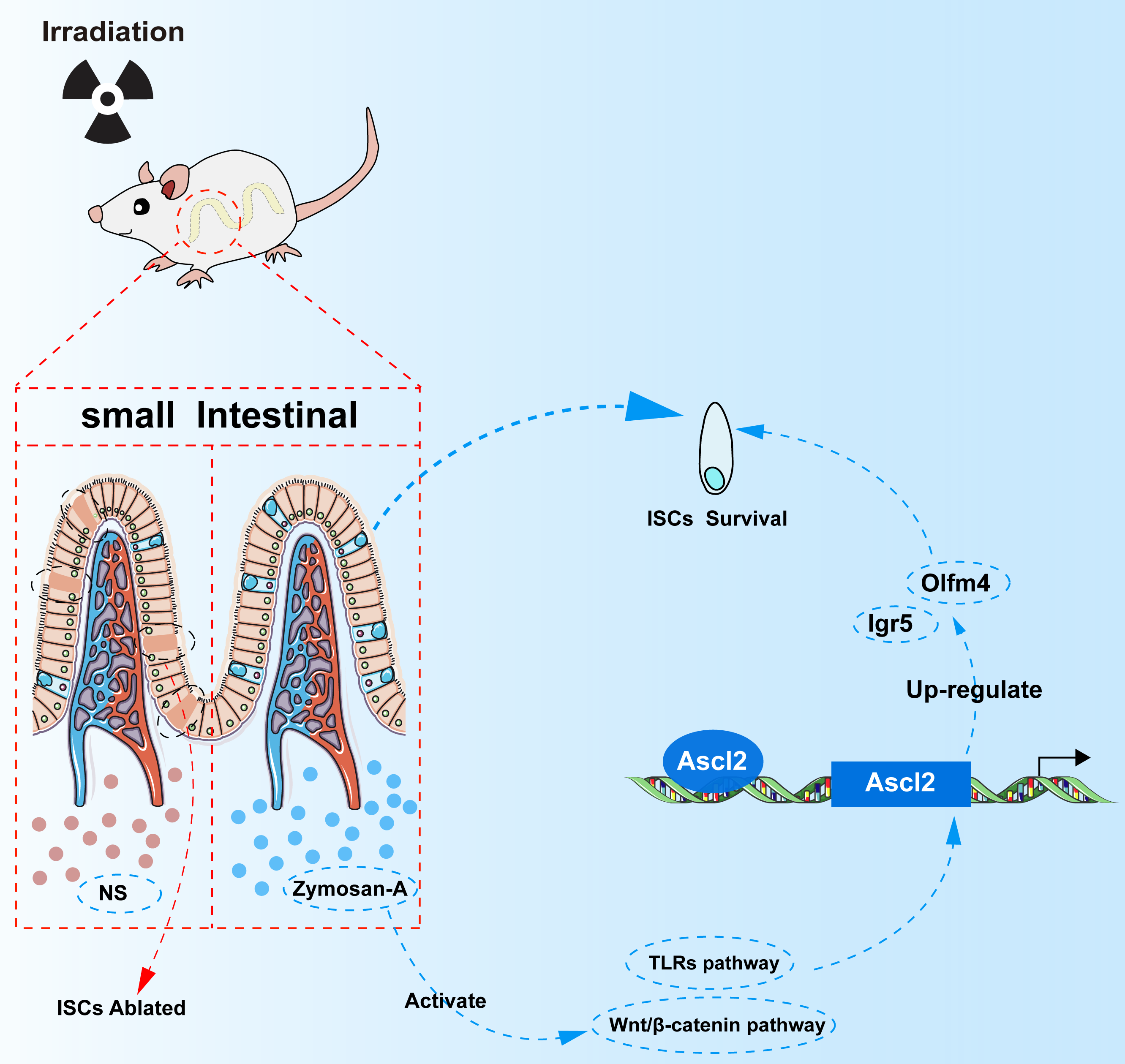

Supplement: Supplementary file 4 — Figure S3. [file 41419_2022_5301_MOESM4_ESM.tif]

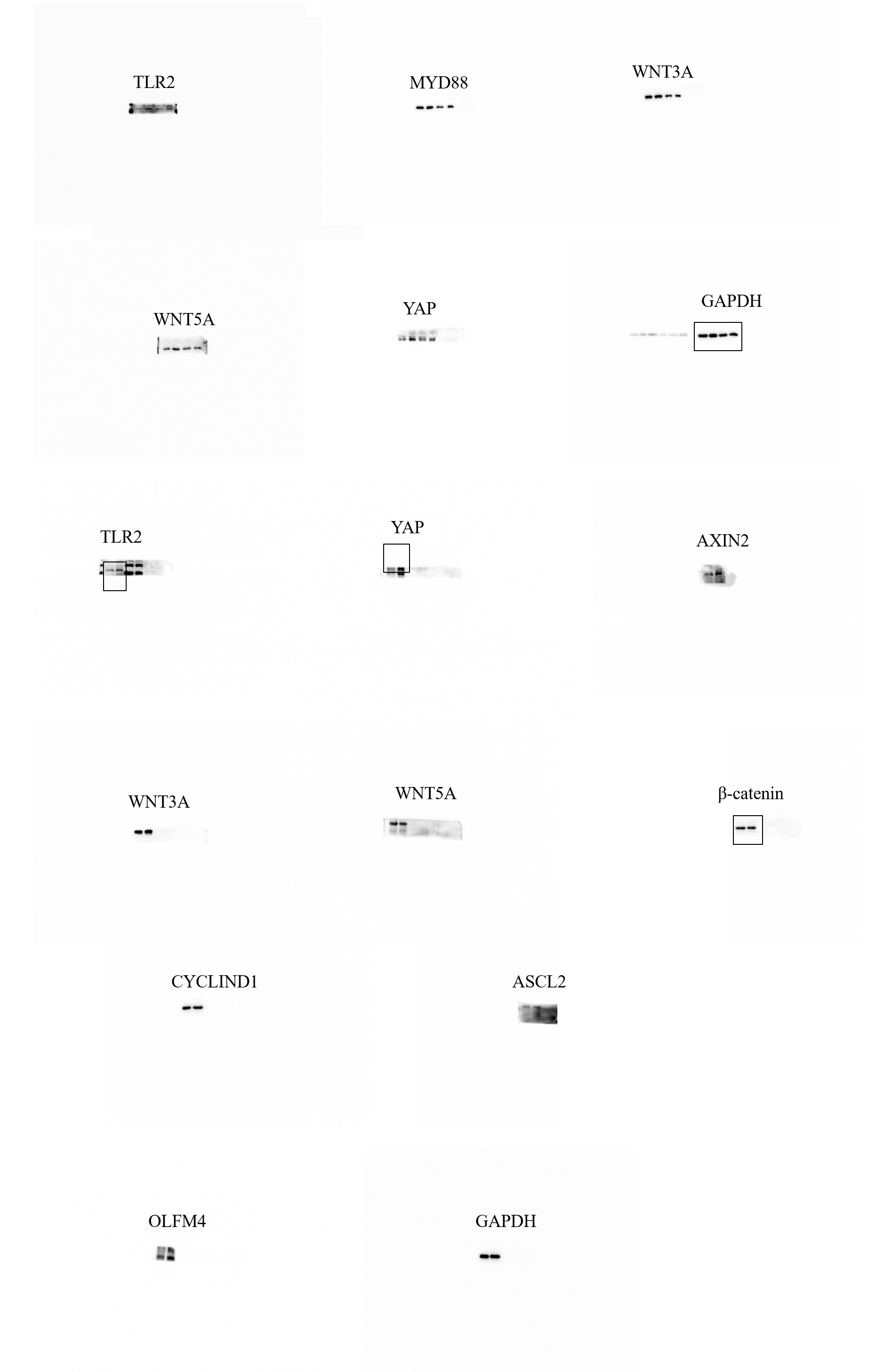

Supplement: Supplementary file 5 — Figure S4. [file 41419_2022_5301_MOESM5_ESM.tif]
